# Community-wide collaboration is a must to reinstall trust in bioinformatics solutions and biomedical interpretation

**DOI:** 10.1515/jib-2022-0028

**Published:** 2023-02-23

**Authors:** Savaş Takan, Jens Allmer

**Affiliations:** Artificial Intelligence and Data Engineering, Faculty of Engineering, Ankara University, Gölbaşı Campus, Ankara, Türkiye; Medical Informatics and Bioinformatics, Institute for Measurement Engineering and Sensor Technology, Hochschule Ruhr West University of Applied Sciences, Mülheim an der Ruhr, Germany

**Keywords:** bioinformatics, code smells, software development, software quality, virtualization

## Abstract

Science has become a highly competitive undertaking concerning, for example, resources, positions, students, and publications. At the same time, the number of journals presenting scientific findings skyrockets while the knowledge increase per manuscript seems to be diminishing. Science has also become ever more dependent on computational analyses. For example, virtually all biomedical applications involve computational data analysis. The science community develops many computational tools, and there are numerous alternatives for many computational tasks. The same is true for workflow management systems, leading to a tremendous duplication of efforts. Software quality is often of low concern, and typically, a small dataset is used as a proof of principle to support rapid publication. Installation and usage of such tools are complicated, so virtual machine images, containers, and package managers are employed more frequently. These simplify installation and ease of use but do not solve the software quality issue and duplication of effort. We believe that a community-wide collaboration is needed to (a) ensure software quality, (b) increase reuse of code, (c) force proper software review, (c) increase testing, and (d) make interoperability more seamless. Such a science software ecosystem will overcome current issues and increase trust in current data analyses.

## 1 Introduction

Today, bioinformatics is arguably involved in any biomedical experiment. Data analysis needs range from applying statistical analyses to very detailed computational studies, for example, in evolutionary biology. With the introduction of sequencing of genomic or transcriptomic sequences, a need for sequence comparison arose. Early tools such as Needleman-Wunsch [1], Smith-Waterman [2], BLAST [3], and FASTA [4], allowed for sequence comparison. Comparisons were either end-to-end (NW) or retrieved subsequences (SW, BLAST, and FASTA). A typical use case involves aligning a handful of short sequences error-tolerantly to more extensive assembled sequences (microbes, RNA transcripts). The demands on such computational tools increased drastically following the advent of massively parallel sequencing (next-generation sequencing). Today gigabytes of short reads need to be aligned to gigabyte-sized genomes. This challenge gave rise to many read aligners, such as Bowtie [5], BWA [6], and STAR [7], to name just a few of the more than 70 available short-read aligners. From the no-free-lunch theorem, we understand that this is a dilemma. On the one hand, we can interpret the abundance as providing us the opportunity to employ the most optimized algorithm for any specific problem in short-read alignment. But on the other hand, we know that all these algorithms perform average when considering all instances of the problem. Thus, this becomes an unfavorable selection problem for the users of such tools. While no comprehensive analysis of these tools has been made compared to pre-microRNA detection algorithms [8], there is also no consistently good documentation, support, or benchmark data. Together, this creates an informed selection, a tour de force for each project involving short read alignment. The complexity of the problem is only further exacerbated by many parameters that need tuning and different input and output data formats.

Biomedical data analysis is, however, not restricted to short-read alignment, and many other data analysis steps are necessary. A similar avalanche of tools is available for any particular task in biomedical data analysis. In proteomics, *de novo* sequencing [9] and database search are examples; the prediction of pre-microRNAs or their targets are examples in post-transcriptional regulation. Considering a large number of available tools, the selection is typically based on *ad hoc* criteria, such as most citations. That can be misleading since earlier tools tend to be cited more. Other measures, such as community around the tool and availability within the data analysis workflow system, are not based on tool correctness but on its outreach. It could be argued that a heavily used tool would be more correct than one that is less used due to the submission of bug reports. However, this opens a new range of issues, including that no feedback mechanism would inform users who previously used the tools of such faults. In the worst-case scenario, wrong information based on erroneous results persists in the knowledge pool. This situation is further aggravated by missing ground truth datasets that could be used to test the tools comprehensively. For example, for a microRNA target sequence prediction, there is no complete description of the mode of biological action so that it could be turned into a correct algorithm. Hence, many solutions are based on the experimentally detected microRNA targets. A guarantee that a particular sequence can under no circumstances act as a miRNA target cannot be provided, though, and, therefore, ground truth data is not available. The same is true for many other research fields where one class (e.g., miRNA target) can easily be observed while the opposite class (e.g., not a transcription factor target) cannot. At least comprehensive testing should be done with synthetic data, but even this is not as simple as it may sound. For example, the fragmentation of peptides within a mass spectrometer’s ion trap is not fully understood and depends on the peptide’s amino acid sequence. Hence, it is impossible to generate a correct synthetic dataset that could comprehensively test *de novo* sequencing or database search algorithms in proteomics.

Taken together, a large amount of not comprehensively tested tools and no available gold standard datasets must lead to unknown result quality. Basing any biomedical decision on such results is dangerous and should only be attempted if many independent approaches support the same explanation. This again puts the load on the users’ shoulders since they have to determine whether some tools’ algorithmic strategies are independent and then integrate their results or invest in many parallel experimental analyses.

FAIR data [10] has become a buzzword and is a fundamental concept for science today. Applying FAIRness to algorithms has been proposed but falls short due to the additional challenges that need to be met and the constant further development of such algorithms. Hence, scientific software needs continuous monitoring and the usage of tools not passing established community tests should be stopped from being published in credible journals. Additionally, a feedback mechanism that questions all previous results should any bugs be found following the release, and the use of a tool needs to be established. This feedback mechanism also doesn’t exist for FAIR data. For example, data fabrication should be communicated to all data consumers, and all findings based on the fabricated data should be put on hold. Our firm belief is that only a community-wide effort can solve the current situation and reinstall trust in scientific analyses and the knowledge they support.

## 2 Analysis environments

As discussed above, a large number of analysis tools are available for many use cases. The same is true for workflow management systems such as Galaxy [11] and KNIME [12]. Not all tools and perhaps not all settings for each tool are available in workflow management tools. However, creating workflows makes data analysis more transparent and should be encouraged. These workflow management tools or any analysis tools, in general, may come with environmental limitations, such as a particular supported operating system and dependencies on other software libraries.

Some differ only in their target operating system or programming language used for their implementation. Whether that is a necessity in light of the availability of virtualization and package management possibilities today is a question that needs to be considered. In the following, we first look at virtualization and then at package management solutions.

### 2.1 Virtualization technology

#### 2.1.1 Virtual machines

Virtualization creates a server, desktop, operating system, file, resource, or network as a virtual resource. One advantage of virtualization is the possibility of partitioning physical computers into several virtual machines that share a common resource. Such virtual machines can provide different runtime environments, such as operating systems (Table 1). Another advantage is that virtual machines can typically create images or snapshots so that this state can be preserved, shared, and executed elsewhere. Today virtualization is widely used, ranging from desktop applications to cloud computing, where a virtual machine may be instantiated many times to solve a computational problem in parallel.

**Table 1:** Some virtual machines and their host and guest operating systems.

Name	Host OS	Guest OS	Price
Microsoft Hyper-V	Windows	Windows, Linux	Free
VMware vSphere	Linux, Windows	Any (>200 OS)	Commercial
Oracle VM	Mac, Windows, Linux, Solaris	Any	Free
QEMU	Any	Any	Free
Red Hat Virtualization	Linux	Linux, Windows	Free
Apple-Boot Camp	Mac	Windows	Free

A Virtual Machine (VM) is a system that simulates the existence of a physical machine. One approach is to split the available resources of a physical device into multiple virtual ones. This allows, for example, running multiple (different) operating systems (OS) on a physical machine. Virtual machines can also be run next to the OS installed on the physical device [2]. Both approaches need a hypervisor that monitors the sharing of resources and VM creation. Popular VM programs are, for example, VirtualBox and VMware. VirtualBox is an open-source virtual machine application that runs on Windows, macOS, and Linux. It has no commercial version and is completely free. The VMware Player is a VM software for Windows and Linux. It is the free equivalent of the retail version of the VMware Workstation.

The opposite, aggregation of physical machines into a large VM (virtualization for aggregation), has also been explored and can be, for example, useful for genome assembly processes, where large amounts of RAM are needed. ScaleMP and Apache Mesos are approached in this direction.

The ability to abstract from physical machines offers the advantage of high efficiency and scalability. New virtual servers can be created very quickly when needed, for example, to create software testing environments or when errors occur. Also, investment in physical machines can be reduced due to load distribution and better use of the hardware capacity. Significant reductions in installation and maintenance have also been observed [13]. Virtualization allows control and reports all currently running servers with centralized management. VMs can be easily transferred to new hardware with the backup taken without losing any application installed in the virtual operating system. Such backups or images prevent the loss of time and information by continuing previous work.

A disadvantage of virtual machines is increased runtime because they cannot access the physical hardware directly. Any hardware problem that may occur will affect all virtual servers. If resource planning, such as CPU and RAM sharing, is not done correctly, severe performance losses can entail.

#### 2.1.2 Container technology

An alternative solution to VMs is Docker. Docker is a new technology that allows development teams to build, manage and secure applications anywhere [14]. Docker and containers are different ways of running software. Docker is an open-source ‘container’ technology [15]. Docker is a technology that provides virtualization thanks to hundreds or even thousands of isolated and independent containers on the same operating system. Thereby, the installation, testing, operation, and deployment of applications are simplified. In addition, it significantly reduces server costs [16, 17].

The Docker daemon is the docker equivalent of a hypervisor for VMs. All the operating system work, such as CPU, RAM, etc., is monitored. The container is the name given to each of the processes run in isolation from each other in the Linux kernel by the Docker Daemon. If we compare Docker to the hypervisor in the VM analogy, the Docker equivalent of each operating system (virtual server) currently running on the physical server is a container. Containers consist of images in layers. A Docker image is all the image files of applications or OSes that are installed in containers. Some containers for database management include MySQL, MongoDB, and MariaDB. There are hundreds of containers available (Table 2). Compose is used to define and run Docker applications with multiple containers [18]. A configuration file is used to compose an application. The outcome is creating and starting all needed services with the configured settings with a single command. Similar to code sharing in GitHub, Docker images are shared on DockerHub.

**Table 2:** Some examples of container technologies.

Name	Type	Publisher	Prices
Docker	Desktop/Server	Docker, Inc.	Open source/License
Linux containers	Desktop/Server	Community	Free
Amazon ECS	A container as a service	Amazon	Pay per use
Red Hat OpenShift	Desktop/Server	Red Hat	License
Microsoft Azure	A container as a service	Microsoft	Pay per use
Google cloud platform	A container as a service	Google	Pay per use

One advantage of Docker is that it does not use a hypervisor and does not host a complete operating system, so it boots in seconds [19]. Docker also stores all the infrastructure requirements of software as code [20]. This leads to versioning which is one of the essential features of Docker. With this feature, the software can be navigated, reproduced, or shared using various service providers. Docker requires fewer resources than a virtual machine, e.g., because it does not include a hypervisor [21]. High traffic demands can be met since thousands of containers are ready within seconds. Development environments differ for each project. Docker puts your applications on a standard footing, making them work the same on every platform.

To manage containers, orchestration solutions are generally used. Since more than one application is running on more than one server in production environments, there is a need for intelligent decisions such as monitoring the resources of the server cluster and running the containers that need to be run on the most suitable server as a source [22]. A container orchestration software is used to make these smart decisions [23]. The main advantages are automatic timing, self-healing abilities, automatic rollout and deployments, load balancing and horizontal scaling, higher resource usage density, functions for business environments, central application management, self-scaling infrastructure, declarative configuration, and reliability. There are many container orchestration tools, such as Kubernetes, Docker Swarm, and Mesosphere.

### 2.2 Package management systems

Package management systems or package managers are systems that ensure the consistent and stable execution of installation, update, configuration, and removal of software packages and libraries. They typically also consider which version of packages and libraries are installed and their dependencies on each other. Most modern package managers have the functionality to download and install software and libraries from a central source. Package management systems (Table 3) can manage software installed on an operating system and install software libraries used during software development and for dependency management.

**Table 3:** Some package management systems.

Name	Aim	Tools	Publisher
DPKG	Debian package management system	APT, APM, synaptic package manager	Debian
RPM	Red Hat package manager	YUM, DNF	Red Hat
NPM	A package manager for the JavaScript	npm	npm, inc
PIP	A package manager for the python	Pip	Community
MAVEN	Build automation tool for JAVA projects	Maven	Apache software foundation
Dart	Use pub get to get dependencies	Pub	Google
C#	Developers share reusable code	NuGet	Microsoft

Package management systems check the correctness and integrity of packages (checksum check) and support digital signatures to authenticate the source of packets. This entails the need for file archiving and decrypting archives which must occur through a central software repository that can allow the software to be updated. Grouping packages according to their types simplifies retrieval. Package managers perform dependency management to ensure that an application is installed, including all dependencies with suitable versions.

Primary package manager operations are package installation/removal, updating processes of packages installed in the operating system, listing installed/not installed packages in the operating system, and managing program dependencies. Operating system package managers are, for example, dpkg, aptitude, and YUM. Software package managers are PEAR, NPM, and Anaconda, to name just a few (Table 3).

Package management systems or package managers are systems that ensure the consistent and stable execution of installation, update, configuration, and removal of software packages and libraries. They typically also consider which version of packages and libraries are installed and their dependencies on each other. Most modern package managers have the functionality to download and install software and libraries from a central source. Package management systems can manage software installed on an operating system and install software libraries used during software development and dependency management.

Package management systems check the correctness and integrity of packages (checksum check) and support digital signatures to authenticate the source of packets. This entails the need for file archiving and decrypting archives which must occur through a central software repository that can allow the software to be updated. Grouping packages according to their types simplifies retrieval. Package managers perform dependency management to ensure that an application is installed, including all dependencies with suitable versions.

Main package manager operations are package installation/removal, updating processes of packages installed in the operating system, listing installed/not installed packages in the operating system, and managing program dependencies. Operating system package managers are, for example, dpkg, aptitude, and YUM. Software package managers are PEAR, NPM, and Anaconda, to name just a few.

## 3 Environmental impact

With virtualization and package managers (see above), running any analysis tool virtually on every system has become possible. Package managers such as chef (https://www.chef.io/) and the node package manager (https://www.npmjs.com/) enable the distribution of an artifact with all its dependencies. They ensure that the versions of the modules on which the artifact depends are compatible and the software functions as desired. Virtualization achieves a similar effect by installing a running system which is then executed in a fixed environment. Both approaches lock the system into a set of artifacts and modules they depend on (state).

As bugs are discovered within libraries or when a module’s functionality changes, the modules’ modifications are not propagated to the virtualized or packaged solution. Deprecated modules and faulty modules can be discovered when creating a virtual machine, Docker image, or package recipe. After deployment, this becomes much more involved and requires a deep understanding of the system, which is a dilemma since these solutions are created to abstract from the system.

A current approach to tool development in science is to build a thesis around a problem that can be solved with a program implemented by the student and perhaps a few supporting younger students. Only very few tools are further developed once the thesis has been defended. Software testing is usually done using a small amount of data measured for a particular purpose. Clearly, such an approach is not in line with modern software testing principles such as early testing, testing for defects, and defect clustering [24, 25]. On the other hand, we need to acknowledge that exhaustive testing is not practically possible, that there is a context dependency for testing, and that problems such as the pesticide paradox and the absence of errors fallacy be considered during testing.

Often the developed artifacts are later re-implemented by other students with a slightly different purpose or taking more information into account for the data analysis. Extension of the previous solution often fails due to unfamiliarity with the code. Other groups develop competing tools with a slight difference in the purpose, solving their particular use case. This leads to a tremendous duplication of efforts.

Why are virtualization and package management not the solutions to this problem? Virtualization and package management guarantee the execution of a fixed set of artifacts. This does not solve the software quality problem and the duplications of efforts but contributes to it. A lock-in is only acceptable if the software has been comprehensively tested, which is rarely possible due to little to no ground truth data available in many areas of basic science. From the systems locked into a running state, it follows that software users are ignorant of bug fixes that might have occurred between the software development and when they apply it to their data analysis task.

This also puts the load of software testing on the users, who need to ensure that the software functions as expected, even with their data. Thus, they don’t only need to deal with sample replicates but also measure data that should be clearly negative or positive to ensure the software system’s functionality. Unfortunately, this is not done in practice. More often than not, work investigating tool quality is ignored due to immense workloads. Data analysis workflows quickly lead to significant computational pipelines, and reviewing each component extensively would be incredibly demanding. In reality, none of the components of current scientific workflows are so dependable that they can be blindly trusted. At the same time, a user of such components is never informed about faults or changes.

In summary, the current situation is highly treacherous and likely leads to misinterpretation of data which, in the best case, only leads to increased laboratory work without outcomes. However, results of faulty analyses may become part of major scientific publications and enter the scientific knowledge pool. The latter can lead to many misdirected efforts, especially if the topic is appealing, such as a gut microbiome–brain connectivity.

## 4 Conclusions

All these problems can easily be solved through community-wide collaboration. All community members can contribute, and the analysis toolbox can grow while remaining non-redundant and at a high technological readiness level [26]. Members may donate their time to algorithm development while others develop tests. Strict reviewing of each solution is required but only needs to be performed if all tests flawlessly pass and no code smells [27] are detected automatically. Typically, any developer in the community can build on existing solutions, and everything is developed once, allowing a better focus on the novel parts that need developing and not the mundane tasks that have been implemented before. Users of the developed solutions can contribute gold standard datasets and develop integration tests that ensure that the software solutions capture the correct scientific question. In summary, a community-driven approach will speed up development, make it more trustworthy, and accelerate scientific discovery and knowledge integration.
